# Variability of the f-cell ratio after treatment of traumatic hemorrhagic shock

**DOI:** 10.1016/j.amsu.2018.10.001

**Published:** 2018-10-05

**Authors:** Faraz A. Khan, Richard Mullins, Anna M. Ledgerwood, Charles E. Lucas

**Affiliations:** a Wayne State University/Detroit Medical Center,USA; b Oregon Health & Science University, USA

**Keywords:** Total blood volume, Body/venous hematocrit ratio, F-cell ratio, Hemorrhagic shock, TBV, Total blood volume, RBCV, Red blood cell volume, PV, Plasma volume, LVHCT, Large venous sample hematocrit, TBHCT, Total body hematocrit, BES, Balanced electrolyte solution, IFS, Interstitial fluid space

## Abstract

**Purpose:**

Measuring total blood volume (TBV) in critically ill patients, using isotope techniques to determine red cell volume (RBCV) and plasma volume (PV) is laborious. Recently, PV measurement using a single bolus dose of tracers has been validated, thus, allowing TBV calculation using large venous hematocrit (LVHCT). However, this technique relies on using a correlation factor, the f-cell ratio, to adjust for differences in LVHCT and total body hematocrit (TBHCT). The normal f-cell ratio is 0.9 but has never been studied in patients recovering from hemorrhagic shock (HS). This study assesses the f-cell ratio at different phases after HS to determine accuracy in calculating TBV.

**Methods:**

114 injured patients requiring immediate operation for HS were studied. All patients had measurements of PV and RBCV via isotope dilution enabling measurements of TBHCT. Correlation of LVHCT and TBHCT were used to calculate the f-cell ratio in the fluid sequestration (n = 81) and in the fluid mobilization period (n = 108).

**Results:**

The f-cell ratio (mean ± SD) averaged 0.89 ± 0.15 and 0.90 ± 0.01 in the first and second halves of the fluid sequestration period versus 0.90 ± 0.2 and 0.80 ± 0.07 in the first and second 48 h of the fluid mobilization period. The f-cell ratio was significantly lower (p=<0.001) in the mobilization period.

**Conclusions:**

These data show for the first time that using PV and LVHCT to calculate TBV after HS is unreliable. The mechanisms causing this variation in f-cell ratio is unknown but likely related to capillary/interstitial dynamics and needs further scientific study.

## Highlights

• Accurate measurements of total blood volume in critically injured patients would be very helpful.
• Direct measurements of total blood volume blood components are very laborious.
• Total blood volume can be estimated using large venous hematocrit and a correction factor.
• The value of f cell ratio is not static during phases of recovery from hemorrhagic shock.
• Variability of f cell ration limits utilization in patients recovering from hemorrhagic shock.

## 1 Introduction

Circulating total blood volume, the sum of red blood cell volume (RBCV) and plasma volume (PV), is an important determinant of cardiac output and organ perfusion [1]. Accurate measurement of TBV requires techniques based on dilution principles where a known quantity of a tracer is injected into a fluid compartment (plasma volume or red blood cell volume) and allowed to equilibrate in that compartment after which the trace concentration is measured, thus, giving the volume of the compartment (volume equals injected tracer/tracer concentration). These methods, however, are arduous requiring accurate tracer injection and frequent blood sampling [2,3], and the relatively long half lives (Cr^51^ = 27.8 days, I^125^ = 60 days, and I^131^ = 8.1 days) of the commonly used tracers preclude frequent repeated measurements [4,5]. Total blood volume, therefore, is usually estimated by surrogates such as central pressures, urine flow, and hematocrit concentrations; these surrogates can be misleading as they are affected by intrathoracic pressure, inotropic efficacy, and compliance of the venous system [6]. Thus, the gold standard method for measuring total blood volume remains indicator dilution.

Recent studies have validated accuracy of the bolus injection of dyes such as indocyanine green for determining plasma volume; this less invasive method can be repeated within short intervals [7]. However, if plasma volume and large venous sample hematocrit (LVHCT) are used to calculate red blood cell volume and, thereby, calculate total blood volume, an inaccurate value is obtained due to the differences in blood flow dynamics between the microvasculature and larger blood vessels (Fahreus-Lindqvist effect) [8]. The difference between total body hematocrit (TBHCT) and large venous sample hematocrit reflects the lower hematocrit in the microcirculation due to the increased velocity of RBC flow through the capillary compared to plasma volume flow. Large venous sample hematocrit, typically, is higher than total body hematocrit when both plasma volume and red blood cell volume are measured directly via dilution techniques. Prior studies have described a near uniform relationship between total body hematocrit and large venous sample hematocrit and have proposed a correction factor, the so called the f-cell ratio (TBHCT/LVHCT), with an estimated value of 0.9 thus, allowing total blood volume calculation when only plasma volume and large venous sample hematocrit values are available. While the f-cell ratio has been studied in a variety of steady states in humans [9,10], this ratio has not been studied in patients recovering from severe hemorrhagic shock. An estimate of the f-cell ratio in this specific subset of patients would therefore be useful to allow accurate measurement of total blood volume. Moreover, any significant differences in the f-cell ratio during the various phases of recovery from severe injury may suggest variations in capillary/interstitial dynamics.

## 2 Material and methods

Following approval by the hospital/university investigation review board (IRB), 114 consecutive severely injured patients, treated on the Emergency Service at Detroit General Hospital, were studied prospectively. The blood volume measurements analysed and reported in this study were performed as part of a multisystem assessment of cardiovascular, pulmonary and renal function following resuscitation from hemorrhagic shock conducted over a period of 3 years and 4 months [11]. The criteria for inclusion in this multisystem study included the need for the patient to receive a minimum of eight red blood cell transfusions during operation if the systolic blood pressure was less than 80 torr or the need to give the patient at least ten red blood cell transfusions if the systolic blood pressure was never below 80 torr. Patients who died on the table or died in the immediate postoperative period were excluded.

These previously obtained but unreported blood volume data were recently analysed due to the recent validation of simpler techniques for plasma volume measurement, thereby, theoretically enabling use of f-cell ratio to calculate total blood volume. All patients received multiple blood transfusions, survived immediate operation for control of bleeding, and were successfully stabilized postoperatively. The resuscitation in transit to the operating room and during operation was designed to correct acute anemia with RBC, restore procoagulants with FFP, and maintain perfusion pressure with balanced electrolyte solution (BES). All of the operations were done by a trauma team headed by a very experienced in-house (24 h) attending trauma surgeon. All of these trauma surgeons were very comfortable operating within the chest, the neck, the abdomen, and the extremities, with extensive experience in obtaining hemostasis from soft tissue injuries and from vascular injuries anywhere in the body. Postoperative support services involved the orthopedic service and the neurosurgical service.

All analyses were stratified for two specific time periods during patient's resuscitation based on specific changes in fluid homeostasis. The early postoperative sequestration phase was defined as the period of obligatory fluid uptake after control of hemorrhage; the duration of this phase was monitored from the end of operation to the time of maximal weight gain. The subsequent mobilization phase represented the period of fluid mobilization and diuresis following maximal weight gain until postoperative weight reached its nadir. The judgment as to when the fluid sequestration period ended and the fluid mobilization period began was based upon serial changes in vital signs, needs, and urine output reinforced by daily weights on a balanced scale; patients with hardware used for the treatment of fractures were not included. The measurements were made during the first and second halves of the fluid sequestration period (equally divided halves) and during the first 48-h and second 48-h period of the mobilization period.

All patients had simultaneous measurements of plasma volume using radioactive iodinated serum albumin (RISA) and red blood cell volume using chromium-tagged (Cr^51^) autologous red blood cells on one or more occasions resulting in a total of 175 observations. All measurements were made by dedicated personnel utilizing the standard methods recommended by the International Committee for Standardization in Hematology [12]. Duplicate samples were collected and disparate results were discarded. Statistical analysis was performed using IBM SPSS Statistics for Windows, Version 21.0. Armonk, NY: IBM Corp.

## 3 Theory/calculation

Total body hematocrit was calculated as RBCV/RBCV + PV, whereas venous blood samples were obtained simultaneously to measure large venous sample hematocrit. The large venous sample hematocrit and total body hematocrit were used to estimate the f-cell ratios after outliers were detected and eliminated using extreme studentized deviate (ESD) test [13]. F-cell ratio in the sequestration and mobilization phases were subsequently compared using student's t-test and ANOVA as appropriate were significance was inferred if p-value <0.05.

## 4 Results

The injury mechanism was a penetrating wound in 106 patients, whereas 8 patients had blunt injury. These patients had an average Injury Severity Score of 25.28 ± 8.3 and ranged in age from 15 to 88 years. There were eight lung injuries included in the 114 patients. Seven of these eight patients also had a laparotomy for control of bleeding, whereas one patient had a very bad extremity wound. Among those with penetrating wounds, 92 were gunshot wounds and 14 were multiple knife wounds. The patients who had penetrating wounds underwent, most commonly, laparotomy (92 patients), thoracotomy (8 patients), or complicated neck operation (3 patients) with the remainder having both laparotomy and thoracotomy. Average vital signs upon arrival included a systolic blood pressure of 74.8 ± 32 torr (SD), pulse rate of 132 ± 46, and an average shock time (systolic blood pressure <80 torr) of 31.4 ± 28 min. During the 7.5 h from admission until the end of immediate operation for control of bleeding, they received an average of 13.9 ± 28 RBC units, 786 ± 657 ml fresh frozen plasma (FFP), and 9.7 ± 4.3 L balanced electrolyte solution. The postoperative period of extravascular fluid sequestration averaged 32.8 ± 25 h during which the patients received an average of 3.5 ± 4 RBC units, 287 ± 538 ml FFP, and 9.3 ± 8.5 L balanced electrolyte solution. The subsequent fluid mobilization period averaged 7.5 ± 7.4 days; during the first four days of this mobilization period, they received an average of 1.5 ± 2.1 RBC units, 39 ± 125 ml FFP, and 12.6 ± 4.8 L balanced electrolyte solution. The f-cell ratio, calculated using 175 simultaneous measurements of total body hematocrit and large venous sample hematocrit, averaged 0.86 ± 0.9. These 175 measurements were obtained during the sequestration period on 68 occasions and during the mobilization period on 96 occasions; one study was done during convalescence. During the sequestration period, the f-cell ratio averaged 0.87 ± 0.09. During the first half of the sequestration phase at an average of 14.2 ± 16 h after operation, the f-cell ratio averaged 0.88 ± 0.1. During the second half of the sequestration period, at an average of 49.4 ± 36.9 h after operation, the f-cell ratio averaged 0.86 ± 0.07. During the fluid mobilization period, at an average of 51 ± 28 h after operation, the f-cell ratio averaged 0.85 ± 0.08. During the first 48 h of the mobilization period at 39 ± 23 h after operation, the f-cell ratio averaged 0.87 ± 0.07. During the second 48 h of the fluid mobilization phase, at an average of 78 ± 18 h after operation, the f-cell ratio averaged 0.80 ± 0.07. The f-cell ratio was significantly higher in the first half of the fluid sequestration period when compared to the second 48 h of the fluid mobilization period (Table 1). The f-cell ratio was significantly higher in the first 48 h of the mobilization period when compared to the second 48 h of the mobilization period (Table 1).

**Table 1:** The F-Cell ratio and postoperative time.

Postoperative Period	First Half Sequestration Period	Second Half Sequestration Period	First 48 Hours Mobilization Period	Second 48 Hours Mobilization Period
Number	47	21	66	30
F-cell Ratio	0.88 ± 0.1^a^	0.86 ± 0.07	0.87 ± 0.007^b^	0.80 ± 0.07
Postoperative Time (Hrs)	14.2 ± 16.0	40.4 ± 36.9	39.0 ± 23.7	78 ± 18.5

a The f-cell ratio was significantly higher during the first half of the sequestration phase when compared to the second 48 h of the mobilization period (p = 0.001).

b Likewise, the f-cell ratio was significantly higher (p = 0.014) during the first 48 h of the mobilization period compared to the second 48 h of the mobilization period.

## 5 Discussion

Rapid identification and interventions aimed to ameliorate or reverse the state of hemorrhagic shock in injured patients remains the corner stone of therapy. Following operative control of active hemorrhage, ongoing monitoring of total blood volume is often helpful in the management of critically ill injured patients as uncompensated hemorrhagic shock can lead to progressive tissue ischemia, acidosis and end organ damage [14]. Due to the logistical constraints associated with direct measurement of total blood volume, clinicians have relied on a variety of indirect parameters such as vital signs and urine output to estimate total blood volume; these parameters often correlate poorly with blood volume changes [6]. The recent validation of tracers such as indocyanine green, used in single bolus doses, to accurately measure plasma volume has afforded clinicians a convenient, repeatable and reliable method to directly measure plasma volume [7]. When total blood volume is calculated from only one of the fractional volumes (plasma volume or red blood cell volume) and large venous sample hematocrit, a correction factor, the f-cell ratio, has to be applied due to the discrepancy between total body hematocrit and large venous sample hematocrit. This difference between total body hematocrit and large venous sample hematocrit is explained by the Fahreus-Lindqvist effect, which reflects the differences in flow dynamics of the RBC in the capillaries as opposed to the larger sized blood vessels. In a capillary where the luminal diameter approximates that of the RBC, the plasma velocity is invariably lower than red cell velocity resulting in a lower hematocrit so that the total body hematocrit is less than the large venous sample hematocrit [15]. Previous investigators have championed near constancy of this correction factor with the f-cell ratio of 0.9 over a wide hematocrit range in healthy adults. Chaplin et al., reported 0.91 ± 0.026 in 19 patients [16]; Berson reported 0.92 ± 0.04 in 25 patients [17]; Gibson et al., reported 0.90 ± 0.082 in 40 patients [18]; and Nachman et al., reported 0.895 ± 0.067 in 40 patients [19]. Thus; measurements of plasma volume can be used to calculate total blood volume [16]. The data, herein, show that this assumption cannot be applied to patients recovering from severe hemorrhagic shock since the f-cell ratio varies according to whether the patient is in the sequestration phase or the mobilization phase.

This variation in the f-cell ratio, likely, is not due to the complexity of the procedures since all collections were made by dedicated personnel and measured in duplicate; disparate results were discarded and not entered into the registry. To the best of our knowledge, these are the only measurements of the f-cell ratio in this subset of patients. The blood volume measurements used in this analysis predated the more recent work aimed at identifying the optimal ratio of blood components for resuscitation following hemorrhagic shock. While the components of the resuscitation regimen are unlikely to alter the blood volume measurements reported herein this could be considered a theoretical limitation of the analysis.

The marked variation in this cohort of patients differs from prior reports. This variability in the f-cell ratio, likely, represents ongoing changes in the capillary/interstitial fluid space dynamics. The Starling Equation identifies that the capillary/interstitial fluid space (IFS) relationship is affected by the oncotic pressure and the hydrostatic pressure within both the capillary and the interstitial fluid space. Although many have assumed that the interstitial fluid space hydrostatic pressure remains constant during various types of insult including hemorrhagic shock, Reed and Rubin showed that the hemorrhagic shock insult was associated with a dramatic and consistent fall in interstitial fluid space hydrostatic pressure [20]. This phenomenon would alter the movement of fluid across the capillary membrane thus favouring the movement of plasma constituents into the interstitial fluid space. This might explain the higher f-cell ratio during the period of postoperative fluid sequestration compared to the later period of fluid mobilization and similarly within the fluid mobilization phase. There are no experimental studies demonstrating how the interstitial fluid space hydrostatic pressure returns to normal but one may assume that this restoration occurs during the later recovery period. Clearly, this phenomenon needs to be evaluated in a controlled experimental model since the therapeutic implications of such a phenomenon are important as it relates to recommendations regarding total blood volume measurements and the role of balanced electrolyte solution and colloid resuscitation for hemorrhagic shock.

## 6 Conclusions

These data show for the first time that using plasma volume and large venous hematocrit to calculate total blood volume after hemorrhagic shock is unreliable. The mechanisms causing this variation in f-cell ratio is unknown but likely related to capillary/interstitial dynamics and needs further scientific study.

## Provenance and peer review

Not commissioned, externally peer reviewed.

## Source(s) of support

None.

## Presentation at a meeting

Organization: Tenth Annual David Fromm Research and Wayne State Surgical Alumni Day.

Place: Wayne State University/Detroit/Michigan.

Date: April 6th, 2016.

## Conflicts of interest

Faraz A. Khan declares that he has no conflict of interest.

Richard Mullins declares that he has no conflict of interest.

Anna M. Ledgerwood declares that she has no conflict of interest.

Charles E. Lucas declares that he has no conflict of interest.

## Registry

Research Registry UIN: researchregistry 4190.

## Ethical approval

Yes, IRB at Detroit General Hospital.

## Sources of funding

None

## Author contribution

Faraz A. Khan, M.D conceptualized and designed the study, performed literature search, reviewed data analysis, prepared the initial draft of the manuscript and approved the final version submitted.

Richard Mullins, M.D. conceptualized and designed the study, performed experimental studies, acquired data, reviewed, edited and approved the manuscript.

Anna M. Ledgerwood, M.D. conceptualized and designed the study, analysed data, reviewed, edited and approved the manuscript.

Charles E. Lucas, M.D. conceptualized and designed the study, performed data analysis reviewed, edited and approved the manuscript.

## Conflicts of interest

None.

## Trial registry number

Research Registry UIN: researchregistry 4190.

## Guarantor

Charles E. Lucas.

## Appendix A Supplementary data

STARD checklist STARD checklist
